# Human trafficking among Ethiopian returnees: its magnitude and risk factors

**DOI:** 10.1186/s12889-019-6395-z

**Published:** 2019-01-22

**Authors:** Lemma Derseh Gezie, Alemayehu Worku Yalew, Yigzaw Kebede Gete

**Affiliations:** 1 Department of Epidemiology and Biostatistics, Institute of Public Health, College of Medicine and Health Sciences, University of Gondar, Gondar, Ethiopia; 2 Department of Preventive Medicine, School of Public Health, College of Health Sciences, Addis Ababa University, Addis Ababa, Ethiopia

**Keywords:** Returnees, Human trafficking, Associated factors, Ethiopia

## Abstract

**Background:**

Human trafficking was affecting a number of individuals in Ethiopia that resulted in various health problems and human right violations. Though the pushing and pulling factors of human trafficking were identified qualitatively, their effect on trafficking status were not measured quantitatively; the magnitude of human trafficking among returnees was not also quantified.

**Methods:**

Primary data were collected from 1342 Ethiopian returning migrants from abroad via Metemma-Yohannes, Moyale, and Galafi border towns from May to October 2016 consecutively. The status of each returnee as trafficked or non-trafficked was determined based on the UN 2000 definition of human trafficking. Factor analyses were conducted on the push and pull factors of migration to identify the underlying constructs. Considering the common underlying concept of items that load on the push and pull factors, the newly emerged construct variables were named in consultation with sociologists before used as independent variables. Finally, the effect of these and other variables on trafficking status were measured using generalized estimation equation.

**Result:**

The magnitude of human trafficking among returning migrants was estimated at 50.89% (95%CI: 0.4822–0.5357). The odds of being trafficked was positively associated with female sex (AOR = 1.55, 95%CI: 1.10–2.17), low household wealth quintile (AOR = 2.55, 95%CI: 1.46–4.44), being smuggled at departure (AOR = 4.48, 95%CI: 3.19–6.29), strong desire for successful oversea life (AOR = 3.98, 95%CI: 2.63–6.02), high level of risk-opportunity imbalance before departure (AOR = 6.10, 95%CI: 4.01–9.30), and strong feeling of hopelessness at success in home-country (AOR = 8.64, 95%CI: 5.62–13.30).

**Conclusion:**

Half of the returned Ethiopian migrants were trafficked. Sex, household wealth quintile, smuggling status, exposure to seductive information about oversea life, risk-opportunity imbalance before departure, and feeling hopelessness for success at home were among the factors associated with human trafficking.

**Electronic supplementary material:**

The online version of this article (10.1186/s12889-019-6395-z) contains supplementary material, which is available to authorized users.

## Background

Human trafficking is currently catching the attention of most governments and internal organizations because of its severe health consequences and subsequent social crisis. It involves the recruitment, transportation, transfer, harboring or receipt of persons usually by force, coercion, or deception for the purpose of exploitation. Exploitation could be apparent in several forms that could include sex trafficking, labor trafficking, child soldiering, and organ removal [1] .

In spite of the difficulty of estimation due to its covert nature, it was estimated that 27 million individuals had been victims of modern day slavery worldwide at any given time because of trafficking [2]. Trafficking in Ethiopia mostly takes the form of transporting migrants by deception, coercion, and then making them susceptible to different forms of exploitation [3]. The 2014 United States report on human trafficking indicated that the number of trafficked and smuggled Ethiopians each day was twice the number of individuals who left the country legally [4].

Unfortunately, because trafficked persons are usually deprived of basic necessities such as food, sleep, hygiene, medical treatment, and safety, they are highly vulnerable to various health problems and human right violations [5]. Though the effects of trafficking could vary depending on the type of exploitation and situations that they underwent, victims were often affected by physical, sexual, psychological, and social impacts. Specifically, due to excessive work load, unsafe and hazardous working conditions, or the use of force by traffickers, victims may develop serious physical health problems. Moreover, as trafficked persons could be exposed to poor and unhygienic living conditions [5] or could cross disease pandemic areas like malaria, they might easily develop infectious diseases, or if they were sex trafficked, they could develop sexually transmitted diseases including human immune-deficiency virus (HIV) and acquired immuno-deficiency syndrome (AIDS) easily. Similarly, because of the high discrepancy between their initial expectation and what they actually encountered at destination, trafficked persons often develop serious mental health problems, including anxiety, depression, and post-traumatic stress disorder (PTSD).

In response to human trafficking and all these and other health consequences of human trafficking as well as the subsequent social crises, governments, non-government organizations (NGOs), and other stakeholders were attempting to fight human trafficking. Despite all those efforts, the problem has not rather been declining if not increasing. Obviously, this should alert researchers as well as governments and organizations combating the problem to recheck the root causes of human trafficking, the relevance of the point of interventions, and the way how it has been implemented.

However, predisposing factors for human trafficking were studied only qualitatively in Ethiopia; neither the effect of those identified factors on human trafficking nor its magnitude was measured quantitatively [6]. Sometimes, human trafficking could be studied by mixing it up with other types of illegal migration. Yet, a qualitatively identified factor without a proper ascertainment of trafficking status, as trafficked or not trafficked, could be unreliable. To fill this identified gap, we conducted a quantitative study on Ethiopian returnees by determining their trafficking status using the United Nations (UN) 2000 definition of human trafficking; the magnitude and the risk factors of human trafficking were determined based on a random sample of Ethiopian returnees.

Studies qualitatively showed the different risk factors for human trafficking, including age. Traffickers prefer children for the purpose of exploitation as younger people usually take emotional decisions that make them vulnerable to peer pressure [7–9]. Authors reported that gender inequalities and disparities were risk factors for women trafficking into prostitution [7]. Poor awareness and lack of formal education were also identified as causes of human trafficking [3, 9, 10].

According to various reports, poverty was a risk factor for human trafficking and could have aspects, like inability to get basic services, insecurity in daily life, and disempowerment in the community that increase vulnerability to trafficking [7, 11–13]**.** Push from parents and relatives, brokers’ deceit that may include false promise of employment at destination and safe travel, as well as false invitations are the other reasons for human trafficking [3, 7] that drive out the poor. On the other hand, there are pulling factors related mainly to the characteristics of receiving countries that facilitate human trafficking. These include cheap labor such as domestic servitude [9, 11] and high demand for commercial sex [11].

Although human trafficking is everywhere, its driving forces might not be identical in all regions as there are variations in socio-cultural, economic, and environmental factors. Therefore, the determinants of human trafficking which are contextually relevant [14] to each individual, relationship, community, and society [15, 16] should be identified for relevant and effective interventions. Studies that could fill these identified gaps and provide input for the formulation of policies and programs to alleviate the problem of human trafficking are highly important. Policies and programs for the new paradigm which considers human trafficking as a public health determinant are also on demand [17]. To this effect, primary data relating to human trafficking were collected from Ethiopians who returned willfully or by deportation, and their status was determined in accordance with the UN 2000 definition of human trafficking. We expect that findings relating to the magnitude of human trafficking and associated factors would contribute to alleviating the existing scanty information [6] and provide inputs for efforts that have already started to curve the problem of human trafficking in the country.

## Methods

### Study setting and period

The current study was conducted at three cross border towns along the three major human trafficking (exit) corridors of Ethiopia, namely Mettema Yohannes, Moyale, and Galafi situated bordering Sudan, Kenya, and Djibouti, respectively. Mettema Yohannes and Moyale are just on points where the Cairo-Cape town High-way enters and leaves Ethiopia, respectively. Galafi is situated on the Ethio-Djibouti border through which the high-way passes. Therefore, all the three towns are the most important gates of human trafficking from all over the country. Individuals who returned to Ethiopia either willfully or by deportation via the three gates were contacted in person and included in the study from May 2016 to October 2016. Returnees might have left Ethiopia through other gates, including Bole International Airport, Bosaso, Humera, and Gambella in addition to the three gates; however, they had to return through the three gates to be included in the study.

### Study design

A quantitative cross-sectional study was conducted to determine the magnitude of human trafficking among Ethiopian returnees and factors associated with it. A pilot study was conducted on 196 returnees (of whom 103 were trafficked persons) at Metemma Yohannes to check the acceptability and applicability of procedures and tools, and to get inputs for sample size determination for the main work.

### Population, sample size, and sampling procedure

The study population consisted of migrants who were abroad seeking better opportunities and returned or would be returned either willfully or by deportation during times closer to the study period. From the pilot study we learned that migrants could recall experiences, situations, and events which happened to them during the past two years. Therefore, Ethiopian migrants whose time of departures from home were during the last 3 to 24 months and returned via the three major trafficking (exit) corridors were included in the study.

Thus, the sample size was determined using the Epi-Info software version 7 for the cross-sectional study with a 1: 1 ratio of exposed to unexposed groups. After comparing the sample sizes determined using different factors in the pilot study, finally, a proportion of human trafficking of 53.48% for junior and lower educational level gave us the maximum sample size. In all cases, a confidence level of 95%, a power of 80%, an odds ratio of 1.5, a contingency of 15% for non-responses and a design effect of 1.5 were assumed. The adequacy of the sample to address the magnitude of human trafficking was checked, assuming an expected proportion of 50%, a margin of error of 5%, and a 95% confidence level. With these assumptions, the sample size was determined to be 1432 returnees. All emigrants coming back home either by deportation or by their own will are usually expected to report to the Ethiopian emigration offices located at the three border towns. Therefore, all returnees who were eligible for the study were interviewed at each check point or in the hotel they booked during the study period until the required sample size was secured.

### Variables

The outcome variable of the study was trafficking status and was leveled as “trafficked” and “non-trafficked”. The trafficking status of each participant was ascertained by interviewing about their age and conditions during their recruitment, travel, and at destination. If a returnee was a child during migration (age less than 18), then by the UN 2000 definition of human trafficking he/she would be considered as trafficked irrespective of consent. Moreover, if a returnee was initially recruited by deception, fraud, or force by brokers or anyone else, and if there was any subsequent exploitation, may be either labor or sexual exploitation or child soldering, then he/she would be considered as a victim of human trafficking [1].

Sometimes, there were situations where traffickers bribe border guards and officials to smuggle migrants. Three or more folds of that money would be the means to control migrants and would be a debit bondage. There were also situations where multiple folds of the money paid by brokers for food, accommodation, transport, etc. was accounted to migrants’ credit. Or brokers could sell migrants to other brokers or traffickers; thus, they could be trapped by the network of continual exploitation unless they escape or give their hands to foreign governments to be deported. In all these and other similar conditions, even though they returned before reaching their destinations, we considered them as victims of human trafficking.

To answer questions about the underlying factors that drive migrants into the trafficking process, data were examined quantitatively using different potential risk factors. These include socio demographic factors (age, sex, marital status, ethnicity, religion, residence, and education), economic factors (household head occupation, household wealth index, family credit pressure before departure), smuggling status when crossing neighboring countries, exit corridor, responsibility to family livelihood, social support, pulling factor (included a questionnaire with 10 items about how people were attracted by foreign conditions), and belief and trust related pushing factor (based on a questionnaire consisting of 11 items that measures the degree of belief and trust people have on their country in changing their life by working at home); trust implies here the confidence they have in home country opportunities, resources, and governance relative to that of their potential destination countries. After conducting factor analyses on the two factors named as ‘pulling factor’ and ‘belief and trust related pushing factor’ (Additional file 1), two underlying factors (components or constructs) were emerged from each.

In consultation with sociologists, after examining the respective items of the puling factor that loaded on each underlying component, the emerged constructs were named as ‘exposure to seductive information about oversea life’ and ‘desire for successful oversea life’. Similarly, considering the belief and trust related pushing factor, the two underlying emerged constructs were named as ‘feeling hopelessness at success in home country’ and ‘risk-opportunity imbalance’. Risk-opportunity imbalance represents the extent returnees under-rate risks that they could face during trafficking and over-estimate opportunities at destination, or it is the extent of outweighing opportunities at destination to risks during trafficking. Thus, the score of constructs named as ‘feeling hopelessness at success in home country’ and ‘desire for successful oversea life’ were each scaled as weak, moderate, and strong by grouping the respective scores. On the other hand, ‘exposure to seductive information about oversea life’ and, ‘risk-opportunity imbalance’ were scaled as low, medium and high.

Human smuggling always involves illegal entry into any other country; thus, individuals involved in this process would be illegal immigrants or criminals [18]. Unlike trafficked persons who would be victims of deception or coercion and continual exploitation [1], smuggled persons would be free from their smugglers control when they reach destinations because their contractual agreement would be to transfer them through international borders up to a certain destination [18]. However, while they are traveling illegally through borders, they could fall in the hands of traffickers, or smugglers could transfer them to traffickers. Therefore, data regarding the smuggling status of each returnee immediately after departure, from Ethiopia to neighboring countries, were collected. However, to minimize the dilemma of its temporal relationship with trafficking, smuggling started at non-neighboring countries or at later times were not considered as far as they were not initially smuggled from Ethiopia to the neighboring countries.

### Data collection tools and procedures

A structured questionnaire was prepared in English (Additional file 2) and Amharic languages to collect data using face to face interview technique. At Metemma Yohannes where most of the returnees were encountered, two male and other two female data collectors, and a field supervisor were assigned. At Moyale and Galafi towns, two data collectors, one from each sex were deployed. Data collectors took training, including field exercise for two days to help them familiarized with the tool. Study participants were interviewed separately by interviewers of similar sex in the waiting rooms of each immigration office or in the hotels they booked. Interpreters were employed when data collectors and respondents did not speak a common language. The principal investigator and field supervisors made on site supervisions during the whole data collection period.

### Statistical analysis

Each copy of the questionnaire filled with data was checked for completeness before it was fed into the computer. The study variables were coded and data were entered into Epi-Info software version 7 and transferred to Stata 14 for analysis (Additional file 3). Descriptive findings including the prevalence of human trafficking by different characteristics of returnees were presented using both texts and tables.

Before making regression analysis, two different groups of questions that were supposed to affect trafficking status [14] were examined using factor analysis. Thus, the first group of questions was about the pulling factor of migration that included 10 items, and the second group constituted the factor related to the degree of belief, attitude, or confidence that individuals had in improving their life by working in their home country. The latter factor which was named as belief and trust related pushing factor consists of 11 questions.

Both eigenvalue above one and scree test criteria were used to identify and retain the meaningful components of each of the two factors that explained much of the variability after varimax (orthogonal) rotation. After rotating the components, decision about factor patterns was based on whether each variable loads 0.40 or more on a component and less than 0.40 on the other components. Thus, variables that load on more than one component were dropped from the analysis. When two constructs emerged after using the principal component analysis, we checked whether both of them were divergently and discriminately valid by observing the correlation matrix, and their reliability was examined with Cronbach’s alpha.

Accordingly, two components each with more than 1 eigenvalue emerged from the factor named as the puling factor. One of the two constructs emerged was named as ‘exposure to seductive information about oversea life’ which represents the extent to which opportunities abroad were advertised to them before departure, and the other construct was named as ‘desire for successful oversea life’ (the level of attraction by materials and quality life that could be maintained or achieved abroad). The reliability test gave a Cronbach’s alpha of 0.84 to the construct named as exposure to seductive information about oversea life and 0.81 to desire for successful oversea life.

In the second factor named as belief and trust related pushing factor, two other components each with eigenvalue greater than one emerged. The construct that emerged first was named to be the level of feeling hopelessness at success in home-country (having more trust and confidence on host-country opportunities than that of home country), and it had a reliability test result of 0.70. The other emerged construct was named as ‘risk-opportunity imbalance’ (the extent of outweighing opportunities at destination to risks during trafficking), and it had a Cronbach’s alpha of 0.80.

Moreover, a composite wealth index was determined by weighting urban and rural specific indexes using the principal component analysis. Some of the questions included in the tool to measure wealth quintile were about the number of people in the household, type of toilet facility, construction material of house, etc. which were adapted from Ethiopian demographic and health survey [19].

To handle the correlation due to clustering of human trafficking by region/zone using a working correlation structure, the Generalized Estimating Equation (GEE) technique was employed so that the marginal effects of variables on human trafficking were determined. Independent variables with *p*-values of 0.20 or less were taken into the multi-variable analysis [20]. The effect of each covariate on the dependent variable was measured by the Adjusted Odds Ratio (AOR), and a p-value of less than 0.05 was considered as statistically significant.

## Result

### Background characteristics

A total of 1342 study participants with a response rate of 93.72% were included in the study. The mean age of the study participants was estimated at 23.95 + 4.55 standard deviations. The youngest and oldest migrants were 14 and 50 years old, respectively. Over half (59.69%) of the participants were males. Of the total participants, about 826 (61.55%) were Muslim, 274 (20.42%) Orthodox, and 223 (16.62%) Protestant by religion. Five hundred sixty-five (42.10%) of the participants were from Oromyia, 332 (24.74%) southern nations, nationalities, and peoples regional state (SNNPRS), and 287 (21.39%) Amhara regions (Table 1).

**Table 1:** Background characteristics of returnees/deported persons via the three major human trafficking corridors, Ethiopia, 2016

Characteristics	Number (percent) of participants
Sex	
Male	801 (59.69)
Female	541 (40.31)
Age	
14–17	43 (3.20)
18–20	251 (18.70)
21–24	477 (35.54)
26–35	466 (33.23)
36–50	125 (9.31)
Religion	
Orthodox	274 (20.42)
Muslim	826 (61.55)
Protestant	223 (16.62)
Others	19 (1.42)
Marital status	
Married	384 (28.61)
Never married	924 (68.85)
Separated/Widowed	34 (2.53)
Region	
Oromyia	565 (42.10)
SNNPRS	332 (24.74)
Amhara	287 (21.39)
Addis Ababa	96 (7.15)
Others	62 (4.62)
Ethnicity	
Oromo	488 (36.36)
Amhara	392 (29.21)
SNNP ^a^	370 (27.57)
Others	92 (6.86)
Educational level	
Illiterate/Informal Education	153 (11.40)
Elementary	417 (31.07)
Junior	353 (26.30)
High school	318 (23.70)
Preparatory school and above	101 (7.53)
Residence (before departure)	
Rural	799 (59.54)
Urban	543 (40.46)
Exit corridor	
Metemma Yohannes	814 (60.66)
Moyale	180 (13.41)
Galafi	252 (18.78)
Others ^b^	96 (7.15)

^a^ SNNP stands for southern nations, nationalities, and peoples, and it comprises more than 40 ethnic groups in SNNPRS; victims of trafficking were mainly from Hadiya, Kembata, Wolayita, and Sidama ethnic groups.

^b^ Other exit gates mainly include Bole Airport, as well as Humera, Bosasso, and Gambella traveling corridors

### Magnitude of human trafficking

Of the total study participants, 683 were found to be victims of human trafficking, yielding a prevalence of 50.89% (95% CI: 48.22–53.57). Three hundred sixty-three (45.32%) of the males and 320 (59.15%) of the females were victims of human trafficking. The prevalence of human trafficking by exit corridor was 387 (47.54%) for Metemma Yohannes, 92 (51.11%) for Moyale, 181 (71.83%) for Galafi, and 23 (23.96%) for other gates (Table 2).

**Table 2:** Human trafficking by background characteristics among returnees/deported persons via the three major human trafficking corridors, Ethiopia, 2016

Characteristics	Magnitude of human trafficking Number (percent)
Sex	
Male	363 (45.32)
Female	320 (59.15)
Age	
14–17	43 (100)
18–20	190 (75.70)
21–24	293 (61.43)
26–35	121 (27.13)
36–50	36 (28.80)
Marital status (before departure)	
Married	130 (33.85)
Never married	533 (57.68)
Separated/Widowed	20 (58.82)
Educational level	
Illiterate/Informal Education	64 (41.83)
Elementary	228 (54.68)
Junior	195 (55.24)
High school	168 (52.83)
Preparatory school and above	28 (27.72)
Residence (before departure)	
Rural	465 (58.20)
Urban	218 (40.15)
Exit corridor	
Metemma Yohannes	387 (47.54)
Moyale	92 (51.11)
Galafi	181 (71.83)
Others ^a^	23 (23.96)

^a^ Other exit gates mainly include Bole Airport, as well as Humera, Bosasso, and Gambella traveling corridors

### Risk factors of human trafficking

After the bi-variable analysis, ethnicity, religion, household head’s occupation, family credit pressure before departure, responsibility for family livelihood, and social support were excluded from the multi-variable analysis as their effects on human trafficking were not significant at 0.20 level of significance. Therefore, after adjusting for other variables, the odds of being trafficked for returnees aged 18–20 was about five times more than that of older migrants aged 30–50 (AOR = 4.65, 95% CI: 2.39–9.05). For females, the odds of being trafficked was 1.55 times (AOR = 1.55, 95% CI: 1.10–2.17) higher than that of males. The odds of being victims of human trafficking for migrants from rural areas was 1.49 times higher than that of migrants from urban areas (AOR = 1.49, 95% CI: 1.03–2.16). The odds of trafficking was more than four-fold for smuggled persons at departure when compared with non-smuggled ones (AOR = 4.48, 95% CI: 3.19–6.29).

Migrants who were moderately exposed to seductive information about oversea life had more than two-fold odds of being trafficked when compared with those who were exposed to such information at a lower level (AOR = 2.32, 95% CI: 1.55–3.48). On the other hand, the odds of being trafficked for migrants who had strong desire for successful oversea life were almost four-fold (AOR = 3.98, 95% CI: 2.63–6.02) times more than that of migrants who had a low level of exposure.

Returnees with a high level of risk-opportunity imbalance before their departures had more than six-fold odds of being trafficked when compared with those who had low level of risk-opportunity imbalance. On the other hand, the odds of being trafficked for returnees who felt strongly hopeless at success in home-country were about 9-fold (AOR = 8.64, 95% CI: 5.62–13.30) more than that of returnees who felt it at a low level (Table 3).

**Table 3:** Factors associated with human trafficking among migrants returned/deported via the three major human trafficking corridors, Ethiopia, 2016

Characteristics	Trafficked (*n* = 1299)		COR(95% CI)	AOR(95% CI)
	Yes	No		
Age (during departure) ^a^				
18–20	190	61	7.70 (4.75–12.48)	4.65 (2.39–9.05)
21–24	293	184	3.94 (2.56–6.04)	2.99 (1.66–5.38)
25–29	121	325	0.92 (0.59–1.43)	0.59 (0.33–1.06)
30–50	36	89	1.0	1.0
Sex				
Male	357	438	1.0	1.0
Female	283	221	1.57 (1.25–1.97)	1.55 (1.10–2.17)
Marital Status				
Married	124	254	1.0	1.0
Never married	96	391	2.60 (2.02–3.34)	1.49 (1.02–2.18)
Divorced/Widowed	20	14	2.93 (1.43–5.99)	2.04 (0.69–6.06)
Educational level				
Illiterate	60	89	1.89(1.09 3.30)	3.01 (1.35–6.70)
Primary school	213	189	3.16 (1.94–5.16)	2.64 (1.33–5.21)
Junior School	187	158	3.32 (2.03–5.45)	2.61 (1.34–5.52)
High school	154	150	2.88 (1.75–4.76)	3.05 (1.53–6.07)
Preparatory and above	26	73	1.0	1.0
Residence before departure				
Rural	434	334	2.05 (1.64–2.57)	1.49 (1.03–2.16)
Urban	206	325	1.0	1.0
Smuggled				
No	121	323	1.0	1.0
Yes	519	336	4.12 (3.21–5.29)	4.48 (3.19–6.29)
Exit corridor				
Metemma Yohannes	355	427	1.0	1.0
Moyale	90	88	1.23 (0.89–1.70)	1.38 (0.85–2.24)
Galafi	174	71	2.95 (2.16–4.02)	4.88 (3.07–7.76)
Others ^b^	21	73	0.35 (0.21–0.57)	0.46 (0.24–0.88)
Household wealth quintile				
Lowest	140	127	2.22 (1.56–3.15)	2.55 (1.46–4.44)
Second	144	101	2.87 (2.00–4.11)	1.85 (1.05–3.26)
Middle	153	123	2.50 (1.77–3.55)	2.47 (1.45–4.20)
Fourth	115	131	1.77 (1.23–2.53)	1.65 (0.97–2.80)
Highest	88	177	1.0	1.0
Exposure to seductive information about oversea life				
Low	183	271	1.0	1.0
Medium	275	159	2.57 (1.95–3.36)	2.32 (1.55–3.48)
High	182	229	1.18 (0.90–1.54)	0.94 (0.61–1.45)
Desire for successful oversea life				
Weak	176	263	1.0	1.0
Moderate	201	242	1.24 (0.95–1.62)	1.73 (1.16–2.57)
Strong	263	154	2.55 (1.94–3.36)	3.98 (2.63–6.02)
Risk-opportunity imbalance				
Low	156	289	1.0	1.0
Medium	203	226	1.66 (1.27–2.18)	1.83 (1.21–2.77)
High	281	144	3.62 (2.73–4.78)	6.10 (4.01–9.30)
Feeling hopelessness at success in home-country				
Weak	148	294	1.0	1.0
Moderate	179	249	1.43 (1.08–1.88)	1.52 (1.02–2.27)
Strong	313	116	5.36 (4.01–7.17)	8.64 (5.62–13.30)

^a^Children aged 14-17 were excluded from the analysis so as not to distort the result because all of them in this group were trafficked according to UN 2000 definition of human trafficking

^b^ Other exit gates mainly include Bole Airport, as well as Humera, Bosasso, and Gambella traveling corridors

## Discussion

The current study identified different characteristics that are contextually relevant for developing countries, analyzed them using the principal component analysis, and determined their association with human trafficking. It showed that almost half of the returning Ethiopian migrants were victims of human trafficking, and this magnitude can be considered as high when compared with other reports [21–23]. It also showed that the trafficking process was associated with socio-demographic factors (age, sex, residence, and educational level), smuggling status, exit corridor, household wealth index, pulling factors (level of exposure to over-advertisement of foreign opportunities and the extent to which they were desiring for successful oversea life), and belief and trust related pushing factor (level of risk-opportunity imbalance and level of hopelessness at success in home-country).

The study demonstrated that young individuals had more tendencies to be trafficked than older ones. This could be justified by the fact that younger persons might not have sufficient prior information about how they could fall in the hands of traffickers and exploiters that could deceive them more easily than older individuals. Nor do younger persons have the maturity to analyze the hidden aims of persons who approach them for the purpose of trafficking. They could also be more easily sold by their ‘care takers’ than older ones. Other studies also showed that younger age facilitates human trafficking [8]. A study on sex workers in Thailand showed that sex trafficked women were younger than their non-trafficked counterparts [21]. Other authors also reported that fear of HIV/AIDS increased the demand for younger girls [8, 24] that could mostly be trafficked.

The current study showed that females were more vulnerable to human trafficking than males. Women and girls might not get sufficient attention at home due to gender inequalities and disparities which could be risk factors that increase their vulnerability to exploitation or trafficking [25]. Women and girls are usually vulnerable to different types of exploitation mainly sexual violence at destinations [26]. Another study done in Ethiopia showed that being female was a guarantee for traffickers to exploit them for a longer period [14]. This is because of the nature of their work, which is mainly housemaid that limits their freedom of movement; as a result, they would not have the opportunity to communicate with others to get out of exploitation. Other authors also reported that women’s documents could be confiscated at destinations and they would be subjected to debit bondage [27]. On the other hand, men and boys might minimize their vulnerability to exploitation as their work which is usually out of home may enable them to meet other people and look for non-exploitive opportunities.

.

Rural residents were at a higher risk of human trafficking than urban dwellers. One possible explanation for this may be the ease to get sufficient information that prevents urban residents from being deceived [14]. For urban residents, it is also easier to get information about how and where to get safer jobs that minimize their exploitation. The bureaucratic process for legal immigration would also be easier for urban residents than rural ones due to their proximity to government offices that process immigration requirements or documents. All these differences could expose rural residents to smuggling, as people usually prefer the swiftest way of migration [3]. Moreover, the focus of brokers and other traffickers is mostly on rural residents because they believe that they are less informed about human trafficking and thus could be easily deceived and lured to overseas. Similarly, other studies showed that one reason why rural migrants frequently trapped by the wave of trafficking was the lack of awareness about trafficking channels [3, 8] which might be better in the case of urban dwellers.

The higher the level of household wealth quintile, the lesser would be the risk of being trafficked. Probably, better economic capacities might have enabled people to process legal requirements needed to go abroad and to take care of threats and possible exploitations. A study done in Ethiopia showed that people from poor families usually wanted to be smuggled into the Middle East instead of going legally. The main reason for developing this belief was that when they went illegally, they wouldn’t take any contractual agreement with any employer before they left their origins. This condition allowed some of them to select jobs with attractive wage rates when they reached safely but illegally at their destinations [14]. However, the problem is that when they go illegally, they could fall in the hands of traffickers; thus, all plans based on uncertain conditions may change their life into tragedy and throw them into continual exploitation.

Another qualitative study done in Ethiopia also showed that households with minimal income initiate women into migration [3], and even though the study didn’t show explicitly its relation with trafficking status, it generally described that poverty forces people into the worst migration conditions. It has also been argued that given the salary scale of workers in the country, irrespective of their educational level, people would fail to lead decent life; as a result, they turn vulnerable to human trafficking [3].

The effect of the level of risk-opportunity imbalance was positively and significantly associated with human trafficking. This is obvious in that if migrants believe that the possibility of getting opportunities is far better than the potential risks, then they might not take sufficient precaution measures that could minimize the risk of deception or fraud and exploitation during migration. Similarly, the level of being hopeless at success in home-country was positively and significantly associated with human trafficking. This may again be true in that if one feels hopeless in his/her home-country with regard to improving his/her welfare, then there could be a possibility of taking any risky decisions, including being smuggled that could usually result in trafficking. A similar finding was reported from another qualitative study done in Ethiopia [14]. As the number of individuals who prefer oversea life to life at home is disproportionately high, then it could negatively affect even community attitude towards changing life by working in country. In this context, a study showed that sending sons and daughters abroad was becoming a symbol of status for some families in Ethiopia [14]; another study done in Nigeria reported a similar finding [27].

The current study showed that returnees who were moderately exposed to seductive information about oversea life would tend to be trafficked more than those who were exposed to a low level of external information from the Media, colleagues, employers, or brokers, etc. However, those who were highly exposed to the advertisement were not different from those who were exposed to a low level of information. Though it was qualitatively, one systematic review reported that being exposed to the features of globalization and getting information about the high demand for labor, which are two of the seven components that constitute the construct named as ‘exposure to seductive information about oversea life’, are reported to be notable factors associated with human trafficking [8]. The absence of a trend association between trafficking status and level of motivating information in the current study may probably be related with a change of migrants’ attitude towards oversea life as they got more and more seductive information; after a certain level of advertisement about oversea life, people may start suspecting the hidden aims of traffickers or brokers that could alert them to take safer measures to prevent themselves from trafficking or minimize the risk of deception for the purpose of exploitation or trafficking.

On the other hand, the current study showed that the stronger the desire for successful oversea life for a person, the more the possibility that he/she would be trafficked. That means, it is rather one of the possibly related characteristics of getting seductive information about foreign life which is the level of desire for successful oversea life that resulted in a trend association with trafficking. This may again imply that beyond getting information about the attractiveness of foreign life if a person is interested in oversea life and opportunities available abroad, then he/she would tend to be trafficked in accordance with the extent to which he/she was attracted by it, no matter whether the stimulating information is genuine or deceptive.

### Limitations

First, the study represents only returnees or those who would return closer in time to the study period. Because it is unclear that the reason why individuals who stay abroad and would not return is related to human trafficking and its consequences, our findings may not be generalized to such segment of the population. Second, migrants who returned by air were not examined quantitatively in this study. Moreover, a returnee might cross the border of a neighboring country without being smuggled, legally; even if he/she was smuggled to cross non-neighboring countries latter, we didn’t consider such returnees as smuggled. This was of course an attempt made to minimize the dilemma of temporality between smuggling and trafficking. Despite these limitations, we identified and quantitatively examined the different risk factors for human trafficking that were contextually relevant for developing countries, like Ethiopia.

### Implications

Evidences showed that human trafficking is a severe form of violence affecting basic human rights and health [17]. Thus, our findings particularly the high prevalence of trafficking among returnees may imply the need to conduct other studies examining the magnitude of subsequent traumatic experiences such as physical, sexual, and psychological violence by recruiting only victims of trafficking. It is also justifiable to conduct similar assessments regarding common health consequences of trafficking such as physical, sexual, and mental health problems; a considerable number of returnees from trafficking in the country might have been suffering from such traumatic events and health outcomes that may need the attention of concerned institutions.

The current study examined socio-demographic and economic factors, smuggling status, exit corridors, and pushing (e.g. feeling hopelessness at success in come-country and risk-opportunity imbalance) and pulling factors (e.g. exposure to seductive information about oversea life and desire for successful oversea life) against trafficking status. However, still there could be a need to understand how these factors are associated to each other concomitantly to affect the trafficking status of individuals. For instance, it is unclear how exposure to seductive information about oversea life is related to a desire for successful oversea life; it is possible that a person who is exposed to an information advertising about oversea life may develop a desire for leading successful life abroad. Conversely, if a person has a desire to lead successful oversea life, then he/she may strive to get information that advertises about opportunities abroad, and vice versa. Thus, to examine this and other similar interwoven relationships among various factors as well as trafficking status, future researchers could employ appropriate statistical methods such as structural equation modeling that examines various variables simultaneously. Because human trafficking is a phenomenon influenced by various factors at individual, relationship/interpersonal, community, and societal levels, it is also worth considering the social ecological model theoretical framework [16] in analyzing its risk factors quantitatively in all these levels using multilevel analysis.

The higher risk of being trafficked for younger group of returnees than the oldest ones could imply the need for paying more attention to minors and younger groups. Because children or younger groups are immature both physiologically and psychologically, the consequences of exploitive and adverse trafficking conditions may be severe among them than older ones. The health seeking behavior of this segment of the trafficked persons may also be poor which could further worsen their health. On the other hand, the high vulnerability of female returnees to be trafficked than male counterparts could suggest that because of disparities in socio-cultural factors, males are relatively in a better condition with regard to the risk of being trafficked not only at home but also in other stages of trafficking after departure. In turn, the disproportionately trafficked women and girls may also be exposed to health problems such as unwanted pregnancy, abortion, and related complications which are unique only to this vulnerable group.

## Conclusion

Half of the returned Ethiopian migrants were trafficked. Risk-opportunity imbalance, being exposed to seductive information about oversea life, feeling hopelessness about success in life at home, and desire for successful oversea life were factors driving migrants into the web of trafficking. Moreover, being smuggled starting from neighboring countries, low educational level and household wealth quintile, young age, and female sex were factors associated with trafficking, and most of these factors could be modified by relevant and context related interventions. Considering the various types of its negative consequences, the high magnitude of trafficking among returnees may imply the need to conduct other studies assessing its subsequent traumatic experiences and health problems.

## Additional files

Additional file 1: Result of factor analysis on pull factors and belief and trust related push factors. It is to identify underlying components that could influence individuals decisional process of migration in unsafe manner that could expose them to human trafficking. (PDF 102 kb)

Additional file 2: Data of Ethiopian returnees after irregular transnational migration. It was about returnees socio-demographic and economic characteristics, trafficking and smuggling status, pull factors, and belife and trust related push factors (i.e. trust in home country resources, opportunities, and governance relative to that of their potential destination countries). (PDF 211 kb)

Additional file 3: English version of the questionnaire. It is a short questionnaire to interview returnees irrespective of their trafficking status and is a part of a long questionnaire that was also employed to interview about the traumatic experiences and mental health symptoms if the returnee is a trafficked person. (XLS 684 kb)

## Abbreviations

AIDS: Acquired Immuno-deficiency Syndrome
AOR: Adjusted Odds Ratio
CI: Confidence Interval
DAAD: German Academic Exchange Service
GEE: Generalized Estimating Eq.
HIV: Human Immune-deficiency Virus
NGOs: Non-government Organizations
PTSD: Post-Traumatic Stress Disorder
SNNP: Southern Nations and Nationalities and Peoples
SNNPRS: Southern Nations and Nationalities and Peoples Reginal State
UN: United Nations
