# Supplementary material for: Human trafficking among Ethiopian returnees: its magnitude and risk factors
Source: BMC Public Health. 2019 Jan 22;19:104. doi: 10.1186/s12889-019-6395-z (PMC6343328; doi:10.1186/s12889-019-6395-z)
Supplement: Supplementary file 1 — Result of factor analysis on pull factors and belief and trust related push factors. It is to identify underlying components that could influence individuals decisional process of migration in unsafe manner that could expose them to human trafficking. (PDF 102 kb) [file 12889_2019_6395_MOESM1_ESM.pdf]

Result of factor analysis on puling and belief and trust related pushing factors to identify underlying components that could influence individuals decisional process of migration in unsafe manner that could expose them to human trafficking

**Name of Tool**	**Item questions considered for each tool**	**Suggested name for the emerged components**	**Cronbach's alpha**
Pull Factor	1. Had you been seduced by your friend or colleague showing life oversea is attractive?<br>2. Were there any promises made by others to attract you and leave home?<br>3. Was there any deception of job promises abroad?<br>4. Were you influenced by globalization (the ease to communicate, cross border, get job, etc.)?<br>5. Was there a demand for labor (may be low skill jobs) in destination areas?<br>6. Was there any promise of lucrative career?<br>7. Were you using TV, radio, Internet or social medias, etc. to get information about overseas?	Exposure to seductive information about oversea life	0.8376
	8. Were you interested in the glamour and anonymity of foreign or modern city life?<br>9. Were you hoping for future fame and success?<br>10. Were there any assets that you were extremely interested in and were out of reach?	Desire for successful oversea life	0.8089
Belief and trust related Push factor	1. For you, it was impossible to do well in business by working in home-country<br>2. It was only abroad which was possible to change and improve your life<br>3. There were no or little opportunities like getting credit to use it as a working capital here in the country and thus it was better to go abroad<br>4. With no or little additional money, what you paid to traffickers (your expense) could be sufficient to be a working capital here in the country*<br>5. Instead of being wealthy here in Ethiopia after many years of effort, it is better to go abroad facing any type of challenge during traveling or on destination	Feeling hopelessness at success in come-country	0.6998
	6. Opportunities from abroad were much greater than possible risks during trafficking/traveling?<br>7. If it was a must to go abroad, there were means available to leave safely*<br>8. The mode of traveling you preferred could enable you to get job abroad without any complications<br>9. Medias exaggerate risks and complications during traveling than possible opportunities abroad<br>10. Instead of broadcasting the success stories abroad, medias communicate the failure ones<br>11. Believe that Ethiopian residents who have a closer relative living abroad (diaspora) lead a better life than people without a diaspora	Risk-opportunity imbalance (Under-rating risks and over-estimating foreign opportunities)	0.7995

*Questions with star were reversed during analyses
