# Supplementary material for: Human trafficking among Ethiopian returnees: its magnitude and risk factors
Source: BMC Public Health. 2019 Jan 22;19:104. doi: 10.1186/s12889-019-6395-z (PMC6343328; doi:10.1186/s12889-019-6395-z)
Supplement: Supplementary file 2 — Data of Ethiopian returnees after irregular transnational migration. It was about returnees socio-demographic and economic characteristics, trafficking and smuggling status, pull factors, and belife and trust related push factors (i.e. trust in home country resources, opportunities, and governance relative to that of their potential destination countries). (PDF 211 kb) [file 12889_2019_6395_MOESM2_ESM.pdf]

## General information

1. ID Number of the respondent _________________
2. Date of interview___________________________
3. Place of interview: a. Metemma Yohannes
   b. Moyale
   c. Galafi

4. Interviewer's name_______________________
   Signature____________Date_____________

5. Supervisor name________________________
   Signature __________ Date______________

| Part I. Socio Demographic and behavioral Characteristics of participants | | | |
|---|---|---|---|
| **S.No** | **Questions** | **Response** | |
| 1.1 | Age at the time of departure | ________________ | |
| 1.2 | Sex | 1.Male 2. Female | |
| 1.3 | Religion | 1. Orthodox 3. Protestant 5. Other specify_________<br>2. Muslim 4. Catholic | |
| 1.4 | Marital Status | 1. Married 2. Never married 3. Divorced 4. Widowed | |
| 1.5 | Ethnicity | 1. Amhara 3. SNNP 5. Somali<br>2. Oromo 4. Tigrie 6. Other (specify)___________ | |
| 1.6 | Educational status | 1. Do not read and write 4. Junior (7th -8th ) 7. Certificate<br>2. Informal education 5. High sch. (9th – 10th) 8. Diploma<br>3. Primary Education 6. Preparatory 9. Degree & above | |
| 1.7 | Former residence | 1. Rural 2. Urban | |
| 1.8 | Former place of residence (Place where you live just before departure due to trafficking) | 1. Region ___________________ Woreda __________________<br>2. Zone______________________ City/town/kebele___________ | |
| 1.9 | At the time of departure, were your parents alive? | 1. Yes, both alive 3. Father only<br>2. Mother only 4. Both not alive | |
| 1.10 | Your own former occupation (if you were independent of your parents) | 1. Job seeker 6. Farming (own or for family)<br>2. Student/ with parents 7. Trade (own or for family)<br>3. Daily laborer 8. Service (own or for family)<br>4. Work for private firms 9. Other (specify)_______<br>5. Government worker | |
| 1.11 | Major means of income for your parents | 1. No parent or care taker 4. Government employee 7. Manufacturing<br>2. Farmer 5. Trade 8. Other (specify)_____<br>3. Work for private firms 6. Service | |
| **Trafficking and smuggling status of respondents** | | | |
| 1.12.1 | **To data collector:** See Question. 1.1 above and check whether the respondent was a child (under 18) during departure. If he/she was a child, ask whether he/she was in an exploitive condition (such as forced labor & services, sex wok, or child soldering) or entering into such a condition. | | 1. Yes<br>2. No |
| 1.12.2 | If you were not a child at departure, how did you enter into the migration process and what were the conditions after departure?<br>**To data collector:** ask whether the returnee was deceived, fraud, cheated or coerced by anyone immediately before or after departure, and was in an exploitive condition (such as forced labor & services, sex wok, or was in military force) or was entering into a similar condition. If positive, choose code 1 (Yes), otherwise code 2 (No) | | 1. Yes<br>2. No |
| 1.12.3 | **To data collector:** See the answer for question number 1.12.1 and 1.12.2. If at least one is "Yes", then the interviewee was a victim of human trafficking. Based on this definition, was he/she trafficked? | | 1. Yes<br>2. No |
| 1.13 | Was a returnee smuggled during traveling?<br>**To data collector:** Check whether the returnee crossed boundaries of neighboring countries illegally (without visa) with voluntary transaction. | | 1. Yes<br>2. No |

| Part II: Other characteristics of participants | | | |
|---|---|---|---|
| **Se. No** | **Questions** | **Responses** | |
| 2.1 | The corridor you travelled in during trafficking | 1. Metemma 3. Galafi 5. Bole<br>2. Moyale 4. Jijiga 6. Other (specify)_______ | |
| 2.2 | Who initiated/ recruited into migration/ | 1. Parents 4. Colleagues 7. Family from abroad<br>2. Other relatives 5. Friend 8. Others (specify)<br>3. Neighbors 6. People from abroad | |
| 2.3 | Do each of the following event happened before departure? If it was positive, write code "1", else code "2". Note: if there was natural disaster, specify it. | 1. Conflict in your surroundings/village ____________<br>2. Conflict in your family ____________<br>3. Political instability ____________<br>4. Economic crisis ____________<br>5. Natural disaster ____________<br>➔ Type of disaster: a) Draught b) Flood c) other(specify)__ | |
| 2.4 | Check whether each of the following event happened? If it was positive, write code "1", else code "2". | 1. Family or yourself were under credit pressure ______<br>2. Denay of your money by traffickers after departure ______<br>3. Falling in debit bondage by traffickers ______<br>4. Forced to pay money by securities during traveling ______<br>5. Forced to pay money by securities during detention ______ | |
| 2.5 | If you were migrant before starting moving abroad, where it was and for what purpose? | 1. Was not migrant<br>2. Around border areas seeking job<br>3. Region/zone/district center seeking job<br>4. Addis Ababa seeking job<br>5. Region/zone/district for education<br>6. Other (specify)________ | |
| 2.6 | Were you a victim of human trafficking before? | 1. Yes<br>2. No | |
| 2.7 | **<u>Only to Females:</u>** Were you forced to marry someone before departure? | 1. Yes<br>2. No | |
| 2.8 | How frequent do you use alcohol **<u>before</u>** and **<u>after</u>** your departure? (Select among 1-5 and write the code on each it applies more) | 1. Before: _______<br>2. After: _______ | 1. I never use alcohol<br>2. Rarely<br>3. 1-4 per week<br>4. Daily<br>5. Others (specify)_______ |
| 2.9 | How frequent do you use drugs other than alcohol **<u>before</u>** and **<u>after</u>** your departure? (Select among 1-5 and write the code on each it applies more) | 3. Before: _______<br>4. After: _______ | 1. Never ➔ go to 2.12<br>2. Rarely<br>3. 1-4 per week<br>4. Daily<br>5. Others (specify)_______ |
| 2.10 | If you were experienced the use of any drug, write the codes in order of frequency | 5. Before: ___ ____ ____<br>6. After: ___ ____ ____ | 1. Kaht 4. Hashish<br>2. Cigar 5. Syringe<br>3. Shisha 6. Other(Specify)______ |
| 2.11 | Had you ever been forced to use any drug by your traffickers (exploiter)? | 1. Never 4. Both alcohol and other drugs<br>2. Yes, substance<br>3. Yes, Alcohol | |
| 2.12 | Had ever been detained/prisoned while you were abroad? | 1. Never<br>2. Yes, in IOM Camp (________ weeks)<br>3. Yes, in Government prisons (________ weeks)<br>4. Others (Specify)________ (_________ weeks) | |

| | | |
|---|---|---|
| 2.13 | At the time of departure, which country was your initial planed destination? | 1. Sudan 4. Europe 7. Other (specify)________<br>2. Djibouti 5. South Africa<br>3. Kenya 6. Other Arab countries |
| 2.14 | What is the main reason for your return now? | 1. Deportation 5. Discomfort with my job 9. Health problem<br>2. Conflict 6. Strict control of borders 10. Other(specify)____<br>3. Visit Family 7. Robe by traffickers<br>4. No job there 8. Insufficient payment |
| **Other social support related questions (OSLO-3)** | | |
| 2.15 | How many people are you so close to that you can count on them if you have great personal problems? | 1. None<br>2. 1-2<br>3. 3-5<br>4. 5 and above |
| 2.16 | How much interest and concern do people show in what you do? | 1. Very little 3. Uncertain 5. A lot<br>2. Little 4. Some |
| 2.17 | How easy is it to get practical help from neighbors if you should need it? | 1. Very difficult 3. Possible 5. Very easy<br>2. Difficult 4. Easy |

| **Part III: A simple poverty scorecard** | | |
|---|---|---|
| **S. N** | **Questions** | **Responses** |
| 3.1 | How many people are in the household? | 1. Six or more 3. Four 5. Two or one<br>2. Five 4. Three |
| 3.2 | Do all children ages 6 to 12 attend school? | 1. Yes 2. No 3. No child aged 6 to 12 |
| 3.3 | Excluding kitchen and toilets, how many rooms does the dwelling unit have? | 1. One 2. Two 3. Three or more |
| 3.4 | What is the main construction material of the walls of the dwelling unit? | 1. Wood and grass, mud and stone, or other<br>2. Wood and mud, reeds and bamboo, cement and stone, hollow blocks. or bricks |
| 3.5 | What type of toilet facility does the household use? | 1. Pit latrine (shared), field/forest, container, etc<br>2. Pit latrine (private)<br>3. Flush toilet (shared)<br>4. Flush toilet (private) |
| 3.6 | What is the main source of cooking fuel? | 1. Firewood (purchase or collected), animal dung<br>2. Crop residue<br>3. Charcoal, kerosene, butane gas, electricity |
| 3.7 | Does the household currently own any mattresses and/or beds? | 1. Yes<br>2. No |
| 3.8 | Does the household currently own any radio? | 1. Yes 2. No |
| 3.9 | Does the household currently own any watches? | 1. Yes 2. No |
| 3.10 | Does the household currently own any cattle, sheep, or goats? | 1. Yes 2. No |
| 3.11 | Does the household currently own any jewelry (gold/silver)? | 1. Yes 2. No |

| Part IV: Puling Factors for Human Trafficking | | |
|---|---|---|
| **SN** | **Question (Considering the situations before departure)** | **Response** |
| 4.1 | Were you interested by the glamour and anonymity of city life? | 1. Yes 2. No |
| 4.2 | Were you hoping for future love, fame, success? | 1. Yes 2. No |
| 4.3 | Had you been seduced by your friend or colleague showing life oversea is attractive? | 1. Yes 2. No |
| 4.4 | Were there any promises made by others to attract you and leave home? | 1. Yes 2. No |
| 4.5 | Was there any deception of job promises abroad? | 1. Yes 2. No |
| 4.6 | Were there any consumer goods that you are interested in and were out of reach? | 1. Yes 2. No |
| 4.7 | Did globalization influence you? | 1. Yes 2. No |
| 4.8 | Was there a demand for labor in destination areas, particularly migrant labor? | 1. Yes 2. No |
| 4.9 | Was there any demand for commercial or transactional sex? | 1. Yes 2. No |
| 4.10 | Was there any promise of lucrative career? | 1. Yes 2. No |
| 4.11 | Are you influenced by TV, radio, Internet to go abroad? | 1. Yes 2. No |
| 4.12 | Are there any societal expectations of children and women to take care of family? | 1. Yes 2. No |

| Part V Belief and trust related push factors and related issues | | |
|---|---|---|
| **S. N** | **Question:** Think about the time before your departure, and answer the following questions | **Response** |
| 5.1 | For you, it was impossible to do well in business by working in home-country | 1. Strongly disagree 3. Neutral 5. Strongly agree<br>2. Disagree 4. Agree |
| 5.2 | It was only abroad which was possible to change and improve your life | 1. Strongly disagree 3. Neutral 5. Strongly agree<br>2. Disagree 4. Agree |
| 5.3 | There were no or little opportunities like getting credit to use as a working capital at home and thus it was better to go abroad | 1. Strongly disagree 3. Neutral 5. Strongly agree<br>2. Disagree 4. Agree |
| 5.4 | With no or little additional money, what you paid to traffickers could be sufficient to be a working capital here in the country* | 1. Strongly disagree 3. Neutral 5. Strongly disagree<br>2. Disagree 4. Agree |
| 5.5 | Opportunities from abroad were much greater than possible risks during trafficking/traveling | 1. Strongly disagree 3. Neutral 5. Strongly disagree<br>2. Disagree 4. Agree |
| 5.6 | If it was a must to go abroad, there were means available to leave safely* | 1. Strongly disagree 3. Neutral 5. Strongly disagree<br>2. Disagree 4. Agree |
| 5.7 | The mode of traveling you preferred could enable you to get job abroad without any complications | 1.Strongly disagree 3. Neutral 5. Strongly disagree<br>2. Disagree 4. Agree |
| 5.8 | Instead of being wealthy here in Ethiopia after many years of effort, it is better to go abroad facing any type of challenge during traveling or on destination | 1.Strongly disagree 3. Neutral 5. Strongly disagree<br>2. Disagree 4. Agree |
| 5.9 | Medias exaggerate risks and complications during traveling than possible opportunities abroad | 1.Strongly disagree 3. Neutral 5. Strongly disagree<br>2. Disagree 4. Agree |
| 5.10 | Instead of broadcasting the success stories abroad, medias communicate the failure ones | 1.Strongly disagree 3. Neutral 5. Strongly disagree<br>2. Disagree 4. Agree |
| 5.11 | Believe that Ethiopian residents who have a closer relative living abroad lead a better life than people without them | 1.Strongly disagree 3. Neutral 5. Strongly disagree<br>2. Disagree 4. Agree |

*The scores are reversed for these items during analysis
